# Targeting Glucose Metabolism in Bladder Cancer: Therapeutic Implications of 2-Deoxy-d-glucose

**DOI:** 10.3390/cimb48100993

**Published:** 2026-09-25

**Authors:** Xia Zhang, Rikiya Taoka, Dage Liu, Akram Hossain, Yuki Matsuoka, Yoichiro Tohi, Hirohito Naito, Kazuya Akimitsu, Ken Izumori, Mikio Sugimoto

**Affiliations:** 1 Department of Urology, Faculty of Medicine, Kagawa University, 1750-1 Ikenobe, Miki-cho 761-0793, Kita-gun, Kagawa, Japan; cho.ka@kagawa-u.ac.jp (X.Z.); akram.hossain@kagawa-u.ac.jp (A.H.); yuchie99@yahoo.co.jp (Y.M.); tohi.yoichiro@kagawa-u.ac.jp (Y.T.); naito.hirohito@kagawa-u.ac.jp (H.N.); sugimoto.mikio@kagawa-u.ac.jp (M.S.); 2 Department of General Thoracic Surgery, Faculty of Medicine, 1750-1 Ikenobe, Miki-cho 761-0793, Kita-gun, Kagawa, Japan; ryu.dagu@kagawa-u.ac.jp; 3 International Institute of Rare Sugar Research and Education, Kagawa University, 2393 Ikenobe, Miki-cho 761-0795, Kita-gun, Kagawa, Japan; akimitsu.kazuya@kagawa-u.ac.jp (K.A.); izumori.ken@kagawa-u.ac.jp (K.I.)

**Keywords:** 2-Deoxy-d-glucose, bladder cancer, glycolysis, hexokinase, oxidative stress, TXNIP, AKT, apoptosis, xenograft model

## Abstract

Bladder cancer is characterized by metabolic reprogramming, and enhanced glycolysis contributes to tumor progression and therapeutic resistance. Because the anticancer activity of 2-Deoxy-d-glucose (2-DG) has been reported in several malignancies, this study evaluated the antiproliferative and metabolism-modulating effects of 2-DG and their association with oxidative stress-related signaling in bladder cancer models. Human bladder cancer cell lines were examined using MTT assays, hexokinase (HK) activity assays, ATP measurements, DCF-DA-based reactive oxygen species (ROS) analysis, and Western blotting. RT112 and 253J cells were selected for mechanistic analyses, and the antitumor efficacy of 2-DG was further evaluated in xenograft models. Treatment with 2-DG reduced the metabolic activity of all five bladder cancer cell lines in a concentration-dependent manner and was associated with decreased HK activity and intracellular ATP levels. Increased ROS-sensitive fluorescence was accompanied by upregulation of TXNIP and BAX and downregulation of AKT and BCL-2, indicating a shift toward a pro-apoptotic pattern of apoptosis-related protein expression. In vivo, systemic administration of 2-DG significantly suppressed xenograft tumor growth without apparent body weight loss or histological abnormalities in the liver or kidneys. These findings suggest that 2-DG suppresses bladder cancer growth in association with metabolic disruption, increased ROS-sensitive fluorescence, TXNIP/AKT-related changes, and modulation of BAX/BCL-2 expression. However, additional studies are required to clarify the underlying mechanisms and further evaluate its safety profile.

## 1. Introduction

Bladder cancer is one of the most common malignancies of the urinary tract and remains a major cause of cancer-related morbidity and mortality worldwide. A substantial proportion of patients experience recurrence, progression to muscle-invasive disease, or treatment resistance despite advances in surgical management, intravesical therapy, systemic chemotherapy, immune checkpoint inhibitors, and targeted therapies. Therefore, therapeutic strategies that address fundamental biological processes involved in bladder cancer progression, including metabolic reprogramming, remain clinically relevant.

Cancer development and progression are closely associated with profound alterations in cellular metabolism that support uncontrolled proliferation, survival, invasion, and resistance to therapy [1]. One of the best-characterized metabolic features of cancer cells is enhanced glycolysis even under normoxic conditions, a phenomenon known as the Warburg effect [2]. Through this metabolic reprogramming, cancer cells preferentially utilize glycolysis for ATP production despite the presence of sufficient oxygen for oxidative phosphorylation. This altered metabolic state enables rapid energy generation and provides essential biosynthetic intermediates required for nucleotide, lipid, and protein synthesis, thereby promoting sustained tumor growth and survival. Increasing evidence suggests that bladder cancer cells also exhibit enhanced glycolytic dependency, making metabolic pathways attractive therapeutic targets.

Among metabolism-targeted therapeutic agents, structurally modified glucose analogs such as 2-Deoxy-d-glucose (2-DG) have attracted considerable attention as promising anticancer compounds [3]. 2-DG differs from glucose by lacking the hydroxyl group at the second carbon position, allowing it to enter cancer cells through glucose transporters and undergo phosphorylation by hexokinase. However, because 2-DG cannot be further metabolized efficiently, it accumulates intracellularly as 2-DG-6-phosphate, resulting in competitive inhibition of hexokinase and disruption of glycolytic flux [4]. Consequently, 2-DG induces ATP depletion, metabolic stress, and impairment of cellular bioenergetics. Cancer cells with high glycolytic dependency are therefore particularly susceptible to 2-DG-mediated cytotoxicity.

In addition to glycolysis inhibition, accumulating evidence indicates that 2-DG can influence cancer cells through several complementary processes, including ATP depletion, altered redox homeostasis, interference with N-linked glycosylation, modulation of autophagy, and changes in apoptosis-related signaling pathways [5,6]. These mechanisms may vary according to tumor type, glucose dependence, drug concentration, exposure duration, and the metabolic context of the cells. Therefore, interpretation of 2-DG-induced decreases in metabolic assays such as MTT requires caution, because reduced signal may reflect cytostatic metabolic suppression rather than direct cell death.

Recent studies have identified thioredoxin-interacting protein (TXNIP) as an important regulator linking cellular metabolism, oxidative stress, and apoptosis. TXNIP negatively regulates the antioxidant thioredoxin system and is induced under conditions of metabolic stress and reactive oxygen species (ROS) accumulation [7]. Increased TXNIP expression has been associated with suppression of cell survival signaling pathways, including AKT signaling, and with apoptosis-related responses in several cancer models [8,9]. However, whether 2-DG-associated metabolic stress is accompanied by TXNIP/AKT-related changes in bladder cancer remains incompletely characterized.

Several studies have reported the antiproliferative activity of 2-DG in a wide range of cancers, including glioma, breast cancer, prostate cancer, and lung cancer. In bladder cancer, prior work has also demonstrated that glycolytic inhibition can suppress tumor growth and may potentiate anticancer therapy [10,11]. Thus, the novelty of the present work does not rest on proposing 2-DG as a completely new anticancer agent. Rather, this study aimed to provide a focused, preliminary characterization of how 2-DG-associated changes in glycolytic stress and ROS-sensitive fluorescence correlate with TXNIP/AKT and BAX/BCL-2 expression in bladder cancer models. Recent studies have further emphasized the relevance of metabolic heterogeneity, glycolysis-associated drug resistance, and oncogenic regulators of glycolysis in bladder cancer [12,13,14,15].

Therefore, the present study aimed to evaluate the antiproliferative and metabolism-modulating effects of 2-DG in bladder cancer. We examined the effects of 2-DG on MTT metabolic activity, hexokinase activity, intracellular ATP production, ROS-sensitive fluorescence, and TXNIP/AKT and apoptosis-related protein expression in multiple human bladder cancer cell lines. In addition, we assessed the in vivo tumor growth-suppressive effect and apparent tolerability of 2-DG using xenograft mouse models. Because the experiments were designed as an initial mechanistic screen, causal interpretation of signaling changes was deliberately limited to associations supported by the available data.

## 2. Materials and Methods

### 2.1. Cell Lines and Culture

Five human bladder cancer cell lines (253J, HT1197, J82, RT112, and T24) were obtained from the Japanese distributor of the American Type Culture Collection (ATCC). Cells were cultured in RPMI-1640 medium (FUJIFILM Wako Pure Chemical Corporation, Osaka, Japan) supplemented with 10% fetal bovine serum (FBS; Thermo Fisher Scientific, Waltham, MA, USA) and 1% penicillin-streptomycin (Thermo Fisher Scientific) and maintained at 37 °C in a humidified incubator with 5% CO_2_.

### 2.2. Cell Viability Assay

Cell viability/metabolic activity was assessed using the 3-(4,5-dimethylthiazol-2-yl)-2, 5-diphenyltetrazolium bromide (MTT) assay. Cells were seeded at a density of 4000 cells/well in 96-well plates and allowed to adhere for 24 h. An initial concentration-screening experiment was performed using the MTT assay. Cells were treated with 2-DG over a broad concentration range of 1–100 mM using six concentrations (1, 5, 10, 25, 50, and 100 mM). Based on the concentration-dependent effects observed in the MTT assay, 10, 25, and 50 mM were selected for subsequent in vitro experiments. Cells were then treated with 2-Deoxy-d-glucose (2-DG; Sigma-Aldrich, St. Louis, MO, USA) at concentrations of 10, 25, or 50 mM for 24 h. Cell viability/metabolic activity was measured using the Cell Proliferation Kit I (Roche, Mannheim, Germany). Absorbance was recorded at 570 nm with a reference wavelength of 750 nm using a microplate reader (BioTek Synergy H1, Winooski, VT, USA). Because MTT is based on cellular reductive/metabolic activity, decreases in MTT signal were interpreted as reduced metabolic activity and proliferative capacity unless supported by additional cell death assays. All experiments were performed in triplicate.

### 2.3. Hexokinase Activity Assay

Hexokinase (HK) activity was measured using the Hexokinase Activity Assay Kit (Cell Biolabs, Inc., San Diego, CA, USA). RT112 and 253J cells were seeded in 6-well plates at a density of 4000 cells/cm^3^ and incubated for 24 h prior to treatment with 2-DG (10, 25, or 50 mM) for an additional 24 h. Cell lysates were prepared according to the manufacturer’s instructions. HK activity was determined by measuring absorbance at 450 nm using a microplate reader. All assays were performed in triplicate.

### 2.4. Cellular ATP Measurement

Intracellular ATP levels were quantified using the ATP Colorimetric Assay Kit (BioVision, Milpitas, CA, USA). RT112 and 253J cells were seeded in 6-well plates at a density of 4000 cells/cm^3^ and treated with 2-DG for 24 h. Following treatment, cells were lysed, and ATP standards and samples were prepared in 96-well plates. After addition of the reaction mixture, plates were incubated for 30 min at room temperature in the dark. Absorbance was measured at 570 nm using a microplate reader. All experiments were performed in triplicate.

### 2.5. Intracellular Reactive Oxygen Species Assay

Intracellular reactive oxygen species (ROS)-sensitive fluorescence was evaluated using 2′,7′-dichlorofluorescein diacetate (DCF-DA; Sigma-Aldrich, St. Louis, MO, USA) and flow cytometry. Cells were seeded at a density of 4000 cells/cm^2^ in 5 cm culture dishes and incubated for 24 h. 2-DG was prepared in sterile PBS or culture medium. DCF-DA stock solution was prepared in methanol, and all samples were exposed to the same final methanol concentration during the DCF-DA staining step. Cells were treated with vehicle control (methanol) or varying concentrations of 2-DG (10, 25, or 50 mM) for 1 h, followed by incubation with 10 μM DCF-DA for 30 min at 37 °C. Fluorescence intensity was analyzed using a CytoFLEX flow cytometer (Beckman Coulter, CA, USA) and CytExpert software, version 2.3 (Beckman Coulter, CA, USA). Because DCF-DA is a general ROS-sensitive probe, the results were interpreted as changes in total ROS-sensitive fluorescence rather than as identification of a specific ROS source.

### 2.6. Western Blot Analysis

After 48 h of 2-DG treatment, cells were harvested and lysed in Cell Lysis Buffer M (FUJIFILM Wako Pure Chemical Corporation, Osaka, Japan) according to the manufacturer’s protocol. Protein concentrations were determined using a bicinchoninic acid (BCA) protein assay kit (Thermo Fisher Scientific, Waltham, MA, USA). Equal amounts of protein (25 µg per lane) were separated by sodium dodecyl sulfate–polyacrylamide gel electrophoresis (SDS-PAGE) on 10% polyacrylamide gels and transferred to polyvinylidene difluoride (PVDF) membranes (Millipore, Burlington, MA, USA). The membranes were blocked with 5% (*w*/*v*) non-fat milk in Tris-buffered saline containing 0.1% Tween-20 (TBS-T) for 1 h at room temperature, followed by overnight incubation at 4 °C with primary antibodies targeting TXNIP (ab188865, Abcam, Cambridge, UK), AKT (ab179463, Abcam, Cambridge, UK), BAX (ab32503, Abcam, Cambridge, UK), BCL-2 (ab182858, Abcam, Cambridge, UK), and GAPDH (FL-335; Santa Cruz Biotechnology, Dallas, TX, USA) as a loading control. The present analysis evaluated total AKT; phosphorylated AKT was not assessed in the current dataset. After washing with TBS-T, membranes were incubated with horseradish peroxidase (HRP)-conjugated secondary antibodies (ab6721; Abcam, Cambridge, UK) for 1 h at room temperature. Protein bands were visualized using a chemiluminescence detection kit (Amersham Biosciences, Buckinghamshire, UK) and imaged with a ChemiDoc MP imaging system (Bio-Rad, Hercules, CA, USA). Densitometric analysis was performed using Image Lab software, version 1.53e (National Institutes of Health, Bethesada, MD, USA). Target protein expression was normalized to GAPDH, and data were expressed as mean ± SD. Statistical analysis was performed using one-way ANOVA followed by Tukey’s multiple-comparison test.

### 2.7. Xenograft Tumor Model in Nude Mice

Male BALB/cA Jcl-nu/nu nude mice (6 weeks old) were obtained from Charles River Laboratories Japan, Inc. (Yokohama, Japan). Male mice were used in this study to reduce potential variability associated with sex-hormone-related factors. All animal experiments were conducted in accordance with the Institutional Regulations for Animal Experiments of Kagawa University (approval no. 19665-1). RT112 and 253J cells (1 × 10^7^ cells) were suspended in 30 µL of collagen (Cellmatrix Type I-A, Nitta Gelatin, Osaka, Japan) and injected subcutaneously into the dorsal flank of each mouse. When tumors reached approximately 100 mm^3^, mice were randomly assigned to two groups (*n* = 6 per group) and treated intraperitoneally with phosphate-buffered saline (PBS) or 2-DG (400 mg/kg) five times per week for 31 days. The 400 mg/kg dose was selected with reference to previous in vivo tumor xenograft studies using comparable doses of 2-DG administered intraperitoneally [16], Tumor size was measured every 4 days using calipers, and tumor volume was calculated using the following formula: tumor volume = length × (width)^2^ × 0.5. At the study endpoint, tumors, kidneys, and livers were excised, fixed in paraffin, sectioned at 4 μm thickness, and stained with hematoxylin and eosin (H&E) for histological evaluation. No serum biochemical toxicity markers or tumor immunohistochemical markers were assessed in the current dataset.

### 2.8. Statistical Analysis

Data are shown as mean ± standard deviation (SD) from ≥3 independent experiments. Normality was confirmed using the Shapiro–Wilk test. Two-group comparisons used Student’s *t*-test, and multi-group comparisons used one-way ANOVA with Tukey’s post hoc test. Longitudinal tumor volumes were analyzed separately for RT112 and 253J xenografts using two-way mixed-design repeated-measures ANOVA, with treatment and time as factors and their interaction included in the model. The Geisser–Greenhouse correction was applied to effects involving time. Post hoc comparisons between treatment groups at individual time points were performed using two-sided Welch *t*-tests with Bonferroni adjustment across the 10 time points. Adjusted *p* < 0.05 was considered statistically significant. Statistical analyses were performed using Python 3.12.14 with NumPy 2.3.5 and SciPy 1.17.0 and GraphPad Prism 9.0.

## 3. Results

### 3.1. Effects of 2-Deoxy-d-glucose on Bladder Cancer Cell Viability

**Figure 1:**
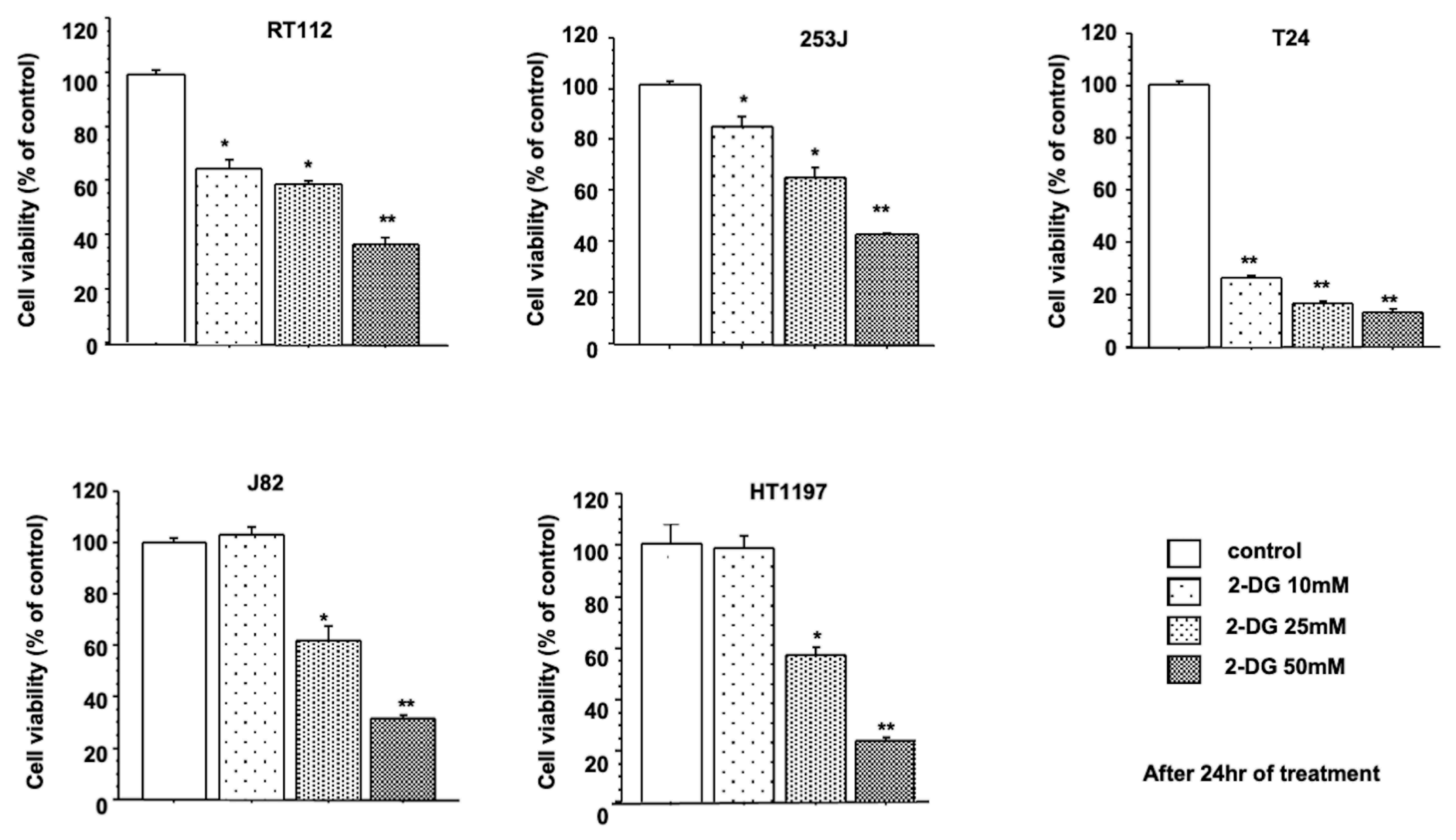
Effect of 2-Deoxy-d-glucose (2-DG) on cell viability in five human bladder cancer cell lines (RT112, 253J, T24, J82, and HT1197). Cells were treated with 10, 25, and 50 mM 2-DG for 24 h, and cell viability was determined using the MTT assay. Data are presented as the mean ± SD of three independent experiments. * *p* < 0.05, ** *p* < 0.01 compared with the untreated control group.

The effects of 2-Deoxy-d-glucose (2-DG) on the MTT metabolic activity of bladder cancer cells (RT112, 253J, T24, HT1197, and J82) were evaluated. As shown in Figure 1, treatment with 2-DG reduced the MTT signal in all five bladder cancer cell lines in a concentration-dependent manner. Because MTT reflects cellular metabolic activity, these findings indicate reduced metabolic activity and/or proliferation rather than direct evidence of cell death.

RT112 and 253J cells exhibited a gradual decrease in MTT signal following treatment with increasing concentrations of 2-DG. Significant reductions were observed at 25 and 50 mM compared with the untreated control group (* *p* < 0.05, ** *p* < 0.01, respectively). Similar inhibitory effects were observed in T24 cells. HT1197 and J82 cells showed greater sensitivity to 2-DG treatment, displaying a marked reduction in MTT signal at 25 and 50 mM. At 50 mM, MTT signal was reduced most prominently in HT1197 and J82 cells compared with the control group (** *p* < 0.01).

Overall, these results demonstrate that 2-DG suppresses MTT metabolic activity in bladder cancer cells in a dose-dependent manner. RT112 and 253J cells were selected for subsequent mechanistic studies because both cell lines showed reproducible responses to 2-DG while maintaining sufficient residual signal at 25–50 mM for biochemical and protein analyses. More sensitive cell lines such as HT1197 and J82 may be useful in future studies focused on determinants of differential metabolic dependency.

### 3.2. 2-Deoxy-d-glucose Inhibits Hexokinase Activity in Bladder Cancer Cells

**Figure 2:**
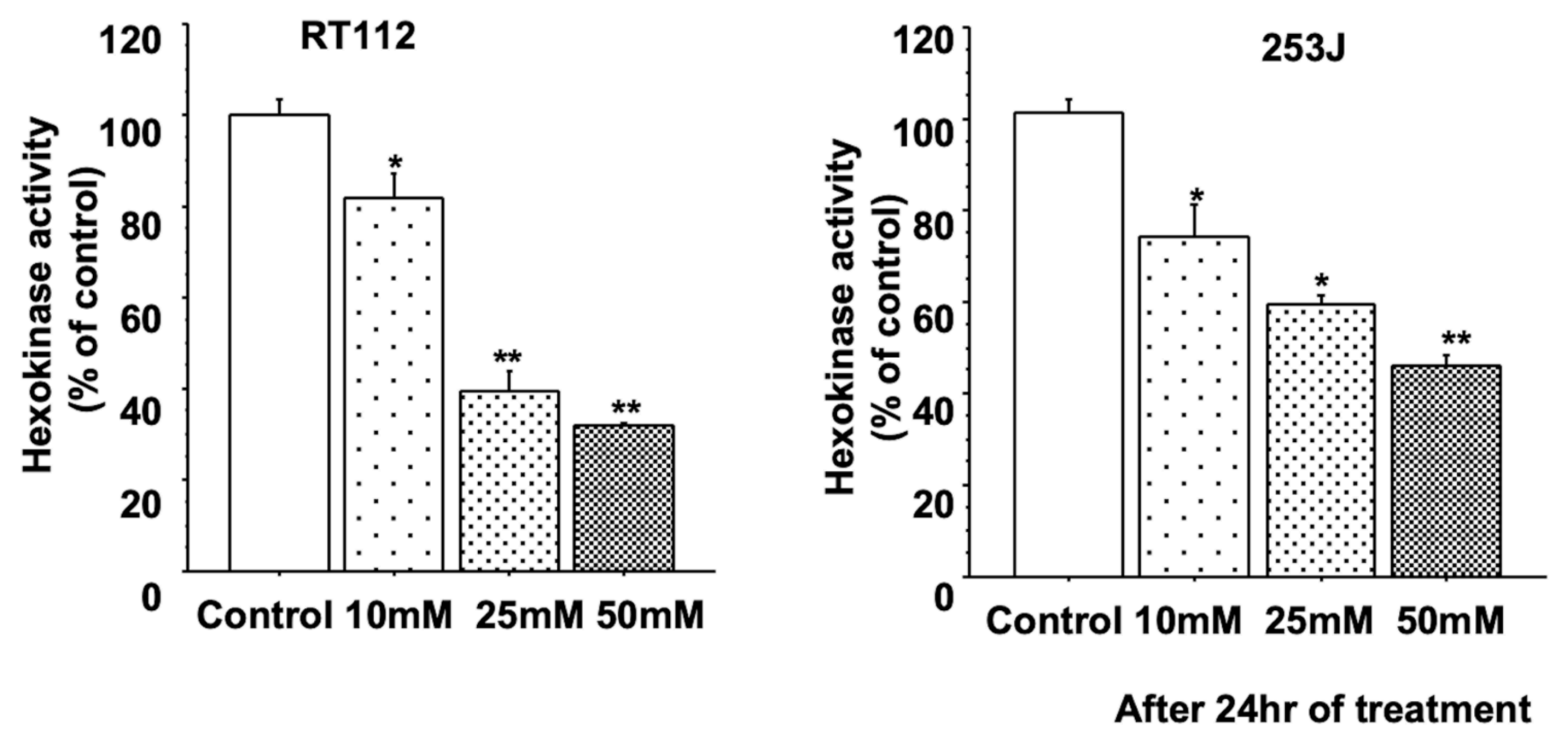
Inactivation of hexokinase (HK) by 2-Deoxy-d-glucose (2-DG) in RT112 and 253J bladder cancer cells. Cells were treated with increasing concentrations of 2-DG (0,10, 25, or 50 mM) for 24 h, and HK activity was measured. Data are presented as mean ± SD of at least three independent experiments, normalized to untreated control cells (set as 100%). Statistical significance: * *p* < 0.05, ** *p* < 0.01 compared to the control group.

To determine whether the antiproliferative effect of 2-DG was associated with altered glycolytic metabolism, hexokinase (HK) activity was examined in RT112 and 253J cells following 2-DG treatment. As shown in Figure 2, treatment with 2-DG reduced HK activity in both cell lines compared with the untreated control group.

The inhibitory effect of 2-DG on HK activity was concentration-dependent, with higher concentrations producing greater suppression. In RT112 cells, treatment with 10, 25, and 50 mM 2-DG reduced HK activity to approximately 80%, 40%, and 32% of control values, respectively. In 253J cells, HK activity was reduced to approximately 76%, 63%, and 50% of control values at the same concentrations. Significant reductions in HK activity were observed following 2-DG treatment (* *p* < 0.05, ** *p* < 0.01 versus control, respectively).

These findings indicate that 2-DG reduces HK activity in bladder cancer cells, supporting an effect on glucose metabolic pathways. However, because direct glycolytic flux markers such as glucose uptake, lactate production, ECAR, OCR, GLUT1, HK2, and LDHA were not measured, the present data should be interpreted as evidence of reduced HK activity rather than a complete characterization of glycolytic flux.

### 3.3. 2-Deoxy-d-glucose Reduces Cellular ATP Levels in Bladder Cancer Cells

**Figure 3:**
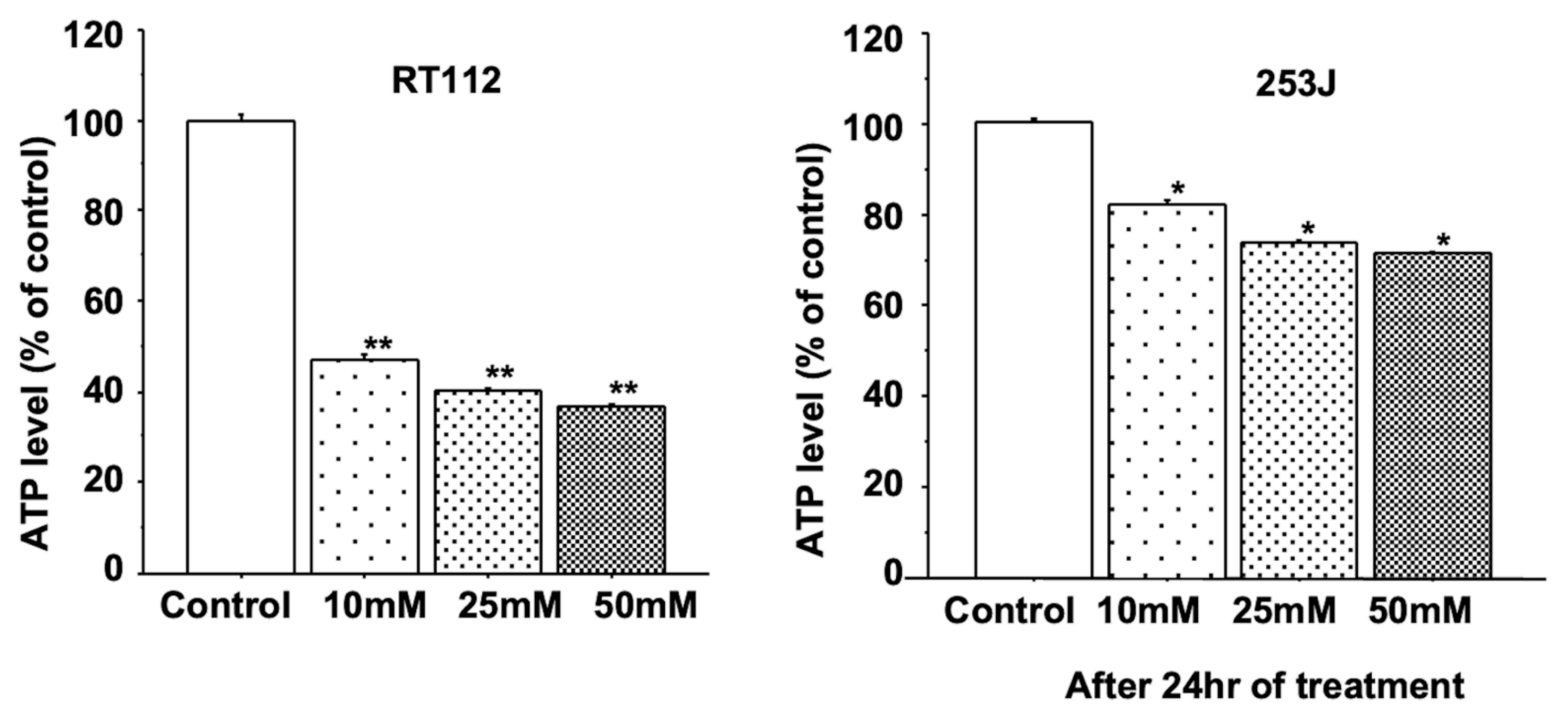
Reduction in cellular ATP levels by 2-Deoxy-d-glucose (2-DG) in RT112 and 253J bladder cancer cells. Cells were treated with increasing concentrations of 2-DG (0, 10, 25, or 50 mM) for 24 h, and intracellular ATP levels were measured. Data are presented as mean ± SD of at least three independent experiments, normalized to untreated control cells (set as 100%). Statistical significance: * *p* < 0.05, ** *p* < 0.01 compared to the control group.

To further investigate the impact of 2-DG on cellular energy metabolism, intracellular ATP levels were measured in RT112 and 253J cells following treatment with 2-DG. As shown in Figure 3, 2-DG reduced intracellular ATP levels in both cell lines compared with the untreated control group, although the magnitude of reduction differed between cell lines.

In RT112 cells, ATP levels decreased markedly after treatment with 2-DG, reaching approximately 40% of control at 25–50 mM 2-DG (* *p* < 0.05, ** *p* < 0.01). In 253J cells, ATP levels were reduced more modestly, remaining approximately 70–80% of control values at the tested concentrations. These findings suggest cell line-dependent differences in energy metabolic response to 2-DG.

The reduction in intracellular ATP levels was accompanied by decreased HK activity and MTT signal. These findings support the conclusion that 2-DG impairs cellular energy metabolism in bladder cancer cells, particularly in RT112 cells, while also highlighting the need for additional metabolic flux assays to define the degree of glycolytic dependence in each cell line.

### 3.4. 2-DG Increases ROS-Sensitive Fluorescence in Bladder Cancer Cells

**Figure 4:**
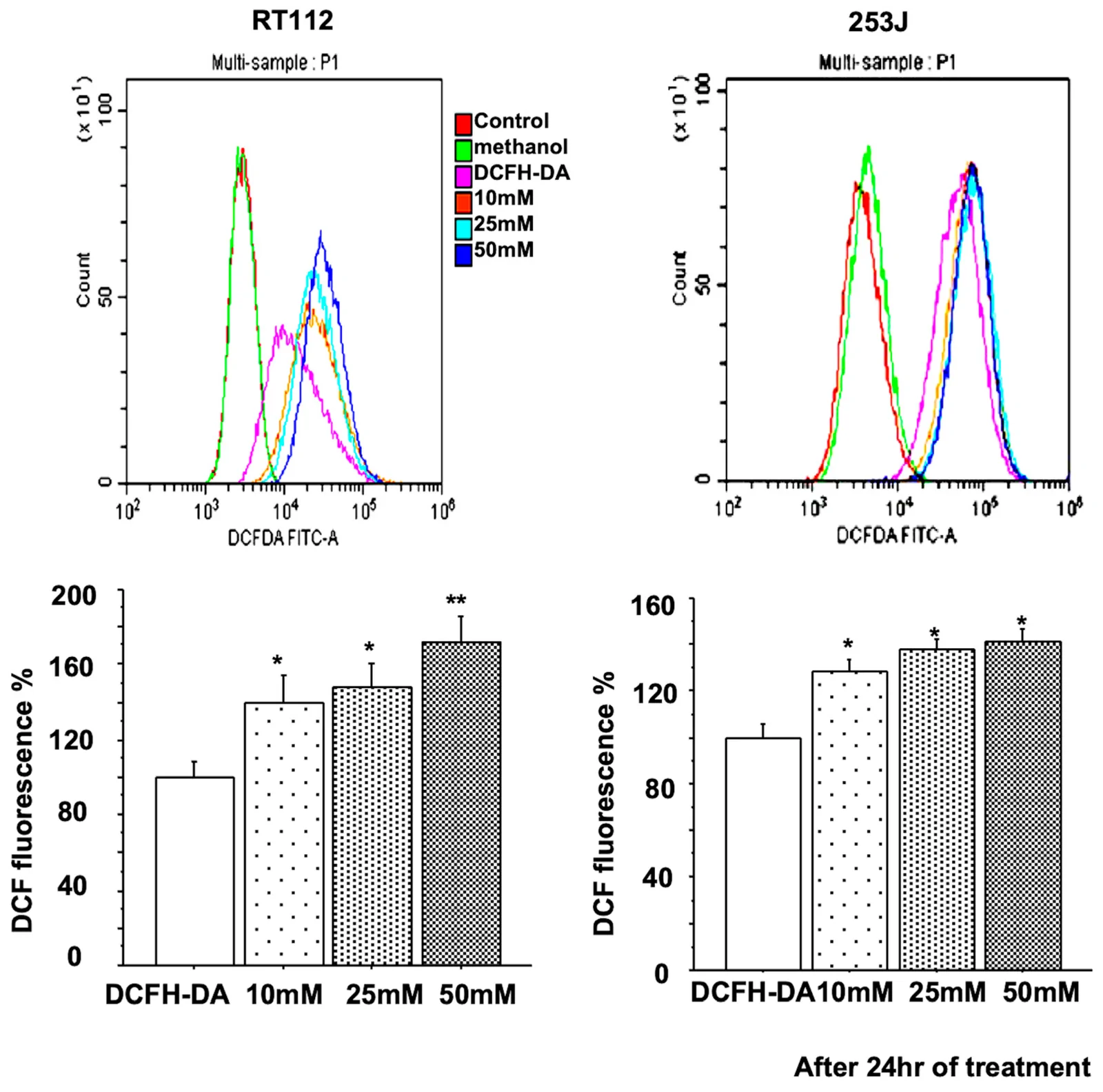
Increase in ROS-sensitive fluorescence by 2-Deoxy-d-glucose (2-DG) in RT112 and 253J bladder cancer cells. Cells were treated with increasing concentrations of 2-DG (0, 10, 25, or 50 mM) for 1 h. Intracellular ROS-sensitive fluorescence was measured using DCF-DA staining followed by flow cytometric analysis. Representative histograms are shown in the upper panels, and quantitative data are presented as mean ± SD of at least three independent experiments, normalized to untreated control cells (set as 100%). Statistical significance: * *p* < 0.05, ** *p* < 0.01 compared with the control group.

To determine whether glycolytic inhibition by 2-DG affects intracellular redox homeostasis, ROS-sensitive fluorescence was measured in RT112 and 253J bladder cancer cells following exposure to 2-DG for 1 h. As shown in Figure 4, treatment with 2-DG increased DCF-DA fluorescence in both cell lines compared with the untreated control group.

Flow cytometric analysis using DCF-DA staining demonstrated a rightward shift in fluorescence intensity following treatment with increasing concentrations of 2-DG, indicating enhanced ROS-sensitive fluorescence. Quantitative analysis confirmed a significant elevation of DCF-DA fluorescence in both cell lines. Because DCF-DA is a nonspecific probe, these results should be interpreted as an increase in total ROS-sensitive fluorescence rather than identification of a specific ROS or subcellular source.

In RT112 cells, DCF-DA fluorescence increased to approximately 150% and 172.1 ± 5.6% of the control at 25 mM and 50 mM 2-DG, respectively (* *p* < 0.05, ** *p* < 0.01). In 253J cells, DCF-DA fluorescence increased more modestly, reaching approximately 140% and 141.0 ± 2.5% of control at the same concentrations (** *p* < 0.01 for both). Thus, ROS-sensitive fluorescence increased in both cell lines, with a more prominent response in RT112 cells.

The increase in ROS-sensitive fluorescence occurred together with reduced ATP levels and decreased hexokinase activity. These findings suggest that 2-DG induces metabolic and redox stress in bladder cancer cells. However, ROS scavenger experiments or mitochondrial ROS-specific probes were not used; therefore, the causal contribution of ROS to reduced MTT signal cannot be established from the present data alone.

### 3.5. 2-DG Is Associated with Changes in TXNIP, Total AKT, and Apoptotic-Related Proteins in Bladder Cancer Cells

**Figure 5:**
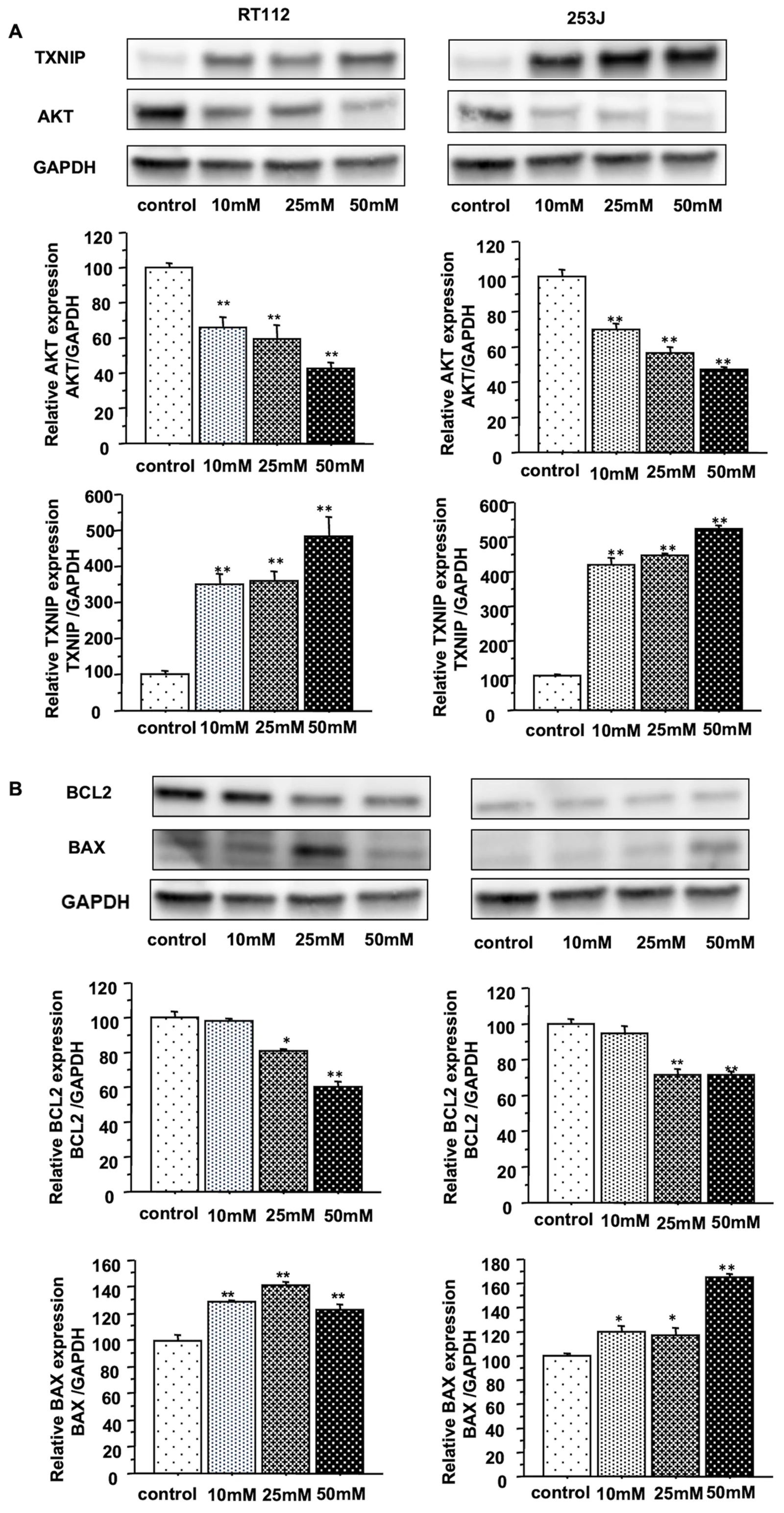
Effects of 2-Deoxy-d-glucose (2-DG) on TXNIP, total AKT, BAX, and BCL-2 expression in RT112 and 253J bladder cancer cells. Cells were treated with increasing concentrations of 2-DG (0, 10, 25, or 50 mM) for 48 h. (**A**) Representative Western blot images showing TXNIP and total AKT, with GAPDH as the loading control. (**B**) Representative Western blot images showing BAX and BCL-2 expression, with GAPDH as the loading control. Densitometric analysis was performed using GAPDH for normalization, and the expression levels were calculated relative to the untreated control. Data are presented as mean ± SD from at least three independent experiments. Statistical significance was determined using one-way ANOVA followed by Tukey’s multiple-comparison test. * *p* < 0.05, ** *p* < 0.01 compared with the control group.

To investigate the molecular changes associated with the growth-inhibitory effects of 2-DG, the expression of TXNIP, total AKT, BAX, and BCL-2 proteins was examined in RT112 and 253J bladder cancer cells following 48 h of treatment with 2-DG. Representative Western blot images and corresponding densitometric analyses are shown in Figure 5A,B. Full-length uncropped Western blot images corresponding to Figure 5 are provided in Figure S1. Protein expression levels were quantified and normalized to GAPDH.

As shown in Figure 5A, 2-DG treatment increased TXNIP expression in both cell lines, with the most pronounced increase observed at 50 mM. In contrast, total AKT expression decreased with increasing concentrations of 2-DG in both cell lines. These findings indicate that 2-DG treatment is associated with changes in TXNIP and AKT protein expression. Because phosphorylated AKT was not measured, the observed reduction in total AKT should not be interpreted as direct evidence of suppression of AKT pathway activity.

Treatment with 2-DG also altered the expression of apoptosis-related regulatory proteins (Figure 5B). BAX expression increased, whereas BCL-2 expression decreased following 2-DG treatment in both RT112 and 253J cells. Consequently, the BAX/BCL-2 ratio was elevated following 2-DG treatment. Densitometric analysis normalized to GAPDH supported these treatment-associated changes in protein expression. These changes indicate a shift toward an apoptosis-associated protein expression profile; however, changes in BAX and BCL-2 expression alone do not execute apoptosis.

The increase in TXNIP expression was accompanied by increased ROS-sensitive fluorescence (Figure 4) and reduced total AKT expression, suggesting an association between metabolic/redox stress and TXNIP and AKT-related changes. TXNIP is known to participate in the regulation of cellular redox homeostasis and can contribute to ROS accumulation [7]. Nevertheless, the present study did not include TXNIP knockdown or overexpression, rescue experiments, or ROS scavenger experiments; therefore, the relationship among 2-DG treatment, ROS, TXNIP, and Akt-related changes should be considered associative rather than causal.

Taken together, these correlative findings indicate that 2-DG is associated with changes in TXNIP, total AKT, BAX, and BCL-2 expression in bladder cancer cells. Further functional experiments, including p-AKT analysis, caspase/PARP cleavage assays, Annexin V/PI staining, cell cycle analysis, and clonogenic assays, are required to determine whether the predominant phenotype is cytostatic, cytotoxic, or context-dependent.

### 3.6. 2-DG Suppresses Tumor Growth in RT112 and 253J Xenograft Models

**Figure 6:**
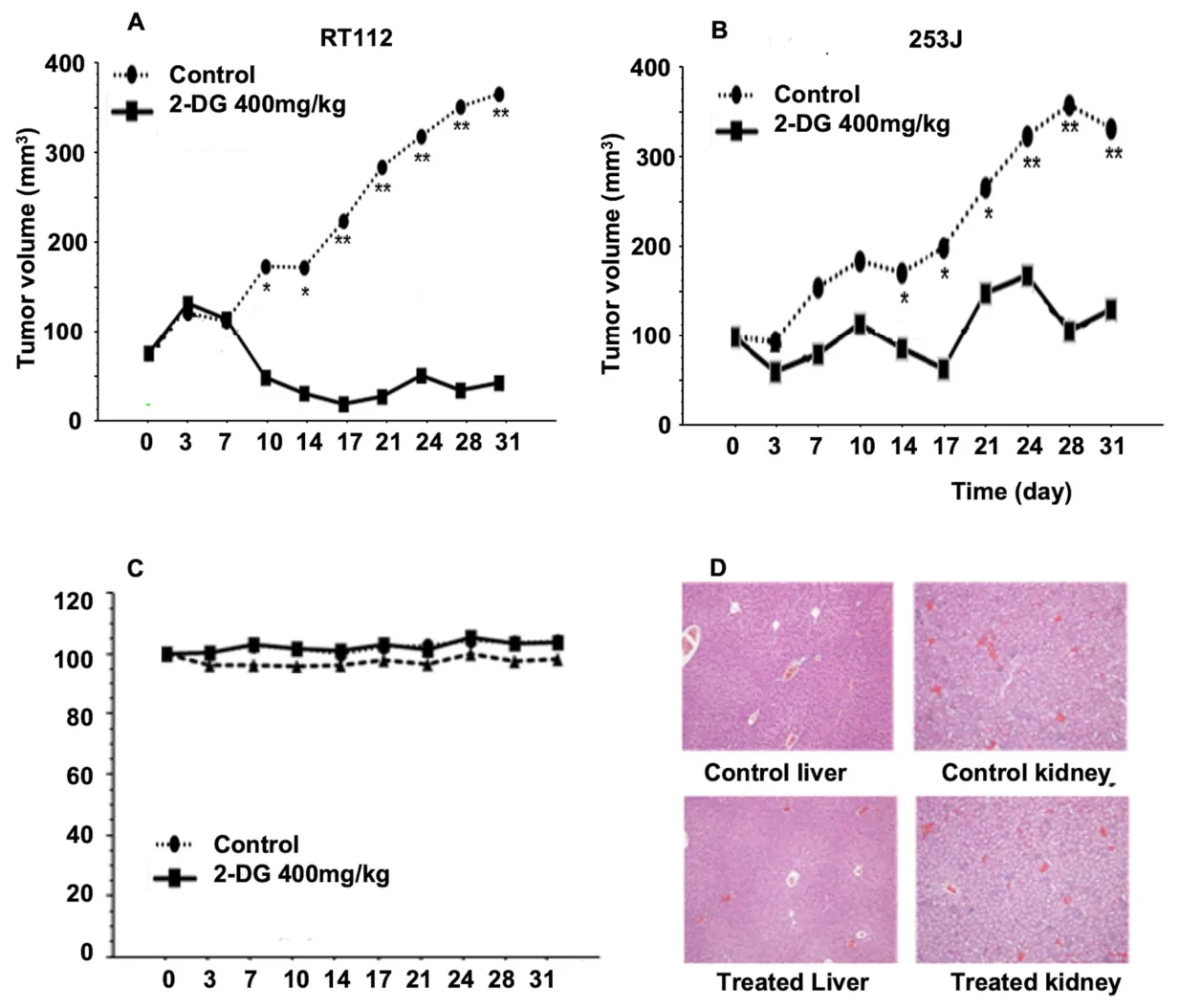
Antitumor effects of 2-Deoxy-d-glucose (2-DG) in bladder cancer xenograft models. RT112 and 253J tumor-bearing nude mice were treated intraperitoneally with vehicle (PBS) or 2-DG (400 mg/kg) five times per week for 31 days. (**A**) Growth curves of RT112 xenograft tumors. (**B**) Growth curves of 253J xenograft tumors. Tumor volumes were measured every 4 days using calipers. (**C**) Body weight changes during the treatment period. (**D**) Representative hematoxylin and eosin (H&E)-stained sections of liver and kidney tissues collected from control and 2-DG-treated mice at the study endpoint (scale bar = 100 μm). Tumor-volume data were analyzed using two-way mixed-design repeated-measures ANOVA with the Geisser–Greenhouse correction for effects involving time. Between-group comparisons at individual time points were performed using two-sided Welch *t*-tests with Bonferroni adjustment across 10 time points. * *p* < 0.05, ** *p* < 0.01 indicate adjusted *p* value.

To evaluate the antitumor activity of 2-DG in vivo, RT112 and 253J bladder cancer xenograft models were established in nude mice. As shown in Figure 6A,B, treatment with 2-DG (400 mg/kg, intraperitoneal injection five times per week for 31 days) suppressed tumor growth in both xenograft models compared with the vehicle-treated control group.

For RT112 xenografts, the main effects of treatment (F (1, 8) = 2.527, *p* = 0.1506), time (F (1.358, 10.865) = 0.817, *p* = 0.4223), and treatment × time interaction (F (1.358, 10.865) = 2.832, *p* = 0.1142) were not significant. Bonferroni-adjusted Welch’s *t*-tests showed no significant differences between groups at any time point (Figure 6A). For 253J xenografts, significant effects of treatment (F (1, 8) = 11.067, *p* = 0.0104) and time (F (1.639, 13.113) = 7.118, *p* = 0.0106) were observed, whereas the treatment × time interaction was not significant (F(1.639, 13.113) = 3.320, *p* = 0.0754). Post hoc Bonferroni-adjusted Welch’s *t*-tests showed a significant difference only at day 8 (adjusted *p* = 0.003758) (Figure 6B). Notably, the inhibitory effect was slightly more pronounced in RT112 tumors than in 253J tumors, consistent with the differential sensitivity observed in vitro (Figure 1).

To assess apparent systemic tolerability, body weight was monitored throughout the study. As shown in Figure 6C, no significant differences in body weight were observed between the control and 2-DG-treated groups during the entire treatment period. No abnormal behaviors or signs of distress were noted in any of the treatment groups.

Histopathological examination of liver and kidney tissues by hematoxylin and eosin (H&E) staining revealed no obvious treatment-related abnormalities in mice receiving 2-DG (Figure 6D). The overall tissue architecture, including hepatocytes, glomeruli, and tubules, appeared comparable to that of the control group. However, serum biochemical analyses such as ALT, AST, BUN, and creatinine were not performed, and no formal histopathological scoring was included.

These findings demonstrate that 2-DG suppresses bladder cancer xenograft growth in vivo with no apparent body weight loss or obvious liver/kidney histological abnormalities observed under the tested conditions. These findings provide preliminary evidence of tolerability; however, additional dose–response, pharmacodynamic, serum biochemical, and tumor tissue analyses are required to define efficacy, mechanism, and safety more comprehensively.

## 4. Discussion

Metabolic reprogramming is a hallmark of cancer and enables tumor cells to sustain proliferation under adverse conditions. Enhanced glycolysis, commonly known as the Warburg effect, is relevant to bladder cancer progression, therapeutic resistance, and tumor heterogeneity [1,2,12,13,14,15,16,17]. Consequently, inhibition of glycolytic metabolism has emerged as an attractive therapeutic strategy. In the present study, we found that the glycolytic inhibitor 2-Deoxy-d-glucose (2-DG) suppresses MTT metabolic activity and xenograft tumor growth in bladder cancer models, with accompanying changes in HK activity, ATP levels, ROS-sensitive fluorescence, and TXNIP/AKT- and BAX/BCL2-related protein expression.

Consistent with previous reports, 2-DG reduced the MTT signal of bladder cancer cells in a concentration-dependent manner [3,4,6,11]. Among the five cell lines examined, RT112 and 253J cells displayed reproducible responses and were therefore selected for mechanistic studies. It is important to note that MTT is a metabolic assay and does not directly distinguish cytostatic effects from cytotoxic cell death. Therefore, the observed decrease in MTT signal should be interpreted primarily as reduced metabolic activity and/or proliferative capacity. The greater sensitivity observed in HT1197 and J82 cells suggests metabolic heterogeneity among bladder cancer cell lines, which should be explored in future studies using a broader molecular subtype panel and normal urothelial controls.

One of the expected effects of 2-DG is interference with glycolytic metabolism. Hexokinase catalyzes the first step of glucose metabolism and is frequently implicated in malignant tumors, including bladder cancer. Our results demonstrated that 2-DG reduced HK activity in both RT112 and 253J cells and decreased intracellular ATP levels, particularly in RT112 cells. However, HK activity and ATP levels alone do not provide a complete picture of glycolytic flux. Direct measurements of glucose uptake, lactate production, extracellular acidification rate (ECAR), oxygen consumption rate (OCR), and expression of GLUT1, HK2, and LDHA will be needed to confirm the degree of glycolytic inhibition and metabolic compensation in each bladder cancer model.

In addition to metabolic inhibition, redox changes may participate in the cellular response to 2-DG. Treatment with 2-DG increased DCF-DA fluorescence in both bladder cancer cell lines, consistent with an increased ROS-sensitive signal. Excessive ROS accumulation can damage cellular macromolecules and activate cell death pathways. Nevertheless, DCF-DA is nonspecific and does not define the ROS source. Moreover, ROS scavenger experiments were not performed. Thus, although the data indicate increased ROS-sensitive fluorescence, they do not prove that ROS is the causal driver of reduced MTT signal or tumor suppression [18].

A notable observation in the present study was the upregulation of TXNIP following 2-DG treatment. TXNIP is a key regulator of cellular redox balance and negatively regulates the thioredoxin antioxidant system. Increased TXNIP expression has been linked to ROS accumulation, oxidative stress, and apoptosis-related responses in various cancer models. In our study, increased TXNIP expression occurred in parallel with increased DCF-DA fluorescence and reduced total Akt expression. However, the causal relationship between TXNIP induction and cellular phenotype was not directly examined. Genetic manipulation of TXNIP or rescue experiments will be required to determine whether TXNIP is a mediator or a marker of 2-DG-induced metabolic stress in bladder cancer cells.

2-DG also reduced total Akt expression in both RT112 and 253J cells. Akt signaling is a major survival pathway that promotes cell proliferation, metabolism, and resistance to apoptosis. However, pathway activity is more directly reflected by phosphorylated Akt and downstream effectors such as mTOR and AMPK [19]. Because only total AKT was evaluated in the present study, the data should be interpreted as a reduction in total AKT abundance rather than direct evidence of AKT pathway inhibition. Future studies should include p-AKT, p-mTOR, AMPK activation, and functional rescue approaches to clarify the role of this pathway.

Our results demonstrated alterations in mitochondrial apoptosis-related proteins, including increased BAX expression, decreased BCL-2 expression, and an increased BAX/BCL-2 ratio. These findings suggest a shift toward a pro-apoptotic protein profile. However, BAX/BCL-2 changes alone do not demonstrate apoptosis execution. Direct assays such as Annexin V/PI staining, caspase-3 and caspase-9 activation, cleaved PARP analysis, and TUNEL staining in xenograft tissues are necessary to confirm whether apoptosis occurs and to determine whether the dominant effect of 2-DG is cytostatic, cytotoxic, or both.

The in vitro findings were supported phenotypically by xenograft studies, in which systemic administration of 2-DG suppressed tumor growth. No significant body weight loss or obvious H&E abnormalities in the liver and kidney were observed under the tested conditions. These data suggest that 2-DG was overtly tolerated in this short-term exploratory model [20,21]. However, safety conclusions should remain cautious because serum biochemical analyses, long-term toxicity studies, dose–response testing, and pharmacodynamic analyses of tumor tissues were not performed. In addition, Ki67, TUNEL, cleaved caspase-3, GLUT1, HK2, and TXNIP/AKT/BAX/BCL-2 validation in tumor tissues would be needed to connect the in vivo tumor growth effect with the proposed mechanism.

Our findings are broadly consistent with previous studies showing that 2-DG suppresses tumor cell growth by interfering with glucose metabolism, although effective concentrations and magnitude of response vary considerably among tumor models. In the present study, 25–50 mM 2-DG produced marked reductions in MTT metabolic activity, HK activity, and intracellular ATP levels, particularly in RT112 cells. Previous studies in bladder cancer and other metabolically active tumor models have also reported growth inhibition and metabolic stress induced by 2-DG, but differences in cell type, exposure conditions, and metabolic dependence make direct comparison of effective concentrations difficult [3,4,6,11]. The increased ROS-sensitive fluorescence observed in our study is also consistent with reports linking 2-DG treatment to metabolic and oxidative stress, although the use of DCF-DA does not establish the source or causal role of ROS [18]. In vivo, the present study showed tumor growth suppression with 2-DG at 400 mg/kg, an intraperitoneal dose within the range used in previous tumor xenograft studies [20]. However, direct comparison of in vitro concentrations and in vivo doses is not appropriate, and the relatively high concentrations used in vitro should be considered exploratory.

Several limitations should be acknowledged. First, mechanistic studies were performed in only two bladder cancer cell lines and did not include normal urothelial cells. Therefore, the present study cannot determine whether the effects of 2-DG are selective for malignant cells or define a therapeutic window. Second, the in vitro concentrations of 2-DG used in this study (25–50 mM) are relatively high and may exceed physiologically achievable plasma concentrations, limiting direct clinical translation. These concentrations were selected based on the initial MTT concentration-screening experiments and should be regarded as an exploratory in vitro range. Third, direct glycolytic flux assays and comprehensive analyses of glycolytic regulators, as well as functional assays of apoptosis, were not performed [22,23]. Fourth, TXNIP, AKT, BAX, and BCL-2 changes were observational and were not functionally validated using knockdown, overexpression, rescue, or inhibitor/scavenger experiments. Fifth, the xenograft study used a single dose level, a limited sample size, and no serum toxicity or tumor pharmacodynamic markers.

Male BALB/cA Jcl-nu/nu mice were used to reduce potential variability associated with sex hormone-related factors. However, the use of only male mice represents a single-sex experimental design and may limit the generalizability of the findings to female animals. In addition, no formal a priori sample-size calculation was performed, and investigator blinding was not used during tumor-volume measurement or histopathological evaluation. These limitations position the present work as an exploratory study that supports further investigation rather than a definitive mechanistic demonstration [24].

Overall, our findings suggest that 2-DG suppresses bladder cancer growth in association with reduced HK activity, ATP depletion, increased ROS-sensitive fluorescence, TXNIP upregulation, reduced total AKT, and modulation of BAX/BCL-2 expression. The data support a model in which 2-DG acts as a metabolic stressor with antiproliferative and tumor growth-suppressive effects in bladder cancer models. Future studies should determine whether these effects are mediated primarily by cell cycle arrest, apoptosis, impaired glycolytic flux, oxidative stress, or context-dependent combinations of these mechanisms, and should evaluate rational combinations with cisplatin, FGFR-targeted therapy, radiotherapy, and immune checkpoint blockade [25].

## 5. Conclusions

The present study demonstrated that 2-Deoxy-d-glucose (2-DG) suppresses bladder cancer cell MTT metabolic activity and xenograft tumor growth in association with reduced hexokinase activity, altered ATP levels, increased ROS-sensitive fluorescence, and changes in TXNIP, total AKT, BAX, and BCL-2 expression. These findings support further investigation of glycolytic metabolism as a therapeutic vulnerability in bladder cancer. However, because direct glycolytic flux measurements, p-AKT analysis, functional apoptosis assays, normal urothelial controls, tumor pharmacodynamic analyses, and comprehensive toxicity studies were not included, the proposed mechanisms should be regarded as correlative and preliminary. Further preclinical studies are required to establish causality, select responsive subtypes, optimize clinically relevant dosing, and evaluate combination strategies.

## Data Availability

The data presented in this study are available from the corresponding author upon reasonable request.

## Supplementary Materials

The following supporting information can be downloaded at: https://www.mdpi.com/article/10.3390/cimb48100993/s1.

## Abbreviations

The following abbreviations are used in this manuscript:

2-DG	2-Deoxy-d-glucose
ATP	Adenosine triphosphate
DCF-DA	2′,7′-Dichlorofluorescein diacetate
ROS	Reactive oxygen species
TXNIP	Thioredoxin-interacting protein
AKT	Protein kinase B (PKB)
BAX	Bcl-2-associated X protein
BCL-2	B-cell lymphoma 2
GAPDH	Glyceraldehyde-3-phosphate dehydrogenase
NF-κB	Nuclear factor-κB
PTEN	Phosphatase and tensin homolog
PBS	Phosphate-buffered saline
