# Supplementary material for: Targeting Glucose Metabolism in Bladder Cancer: Therapeutic Implications of 2-Deoxy-d-glucose
Source: Curr Issues Mol Biol. 2026 Sep 25;48(10):993. doi: 10.3390/cimb48100993 (PMC13703226; doi:10.3390/cimb48100993)
Supplement: Supplementary file 1 [file cimb-48-00993-s001.zip › cimb-4518854-supplementary.pdf]

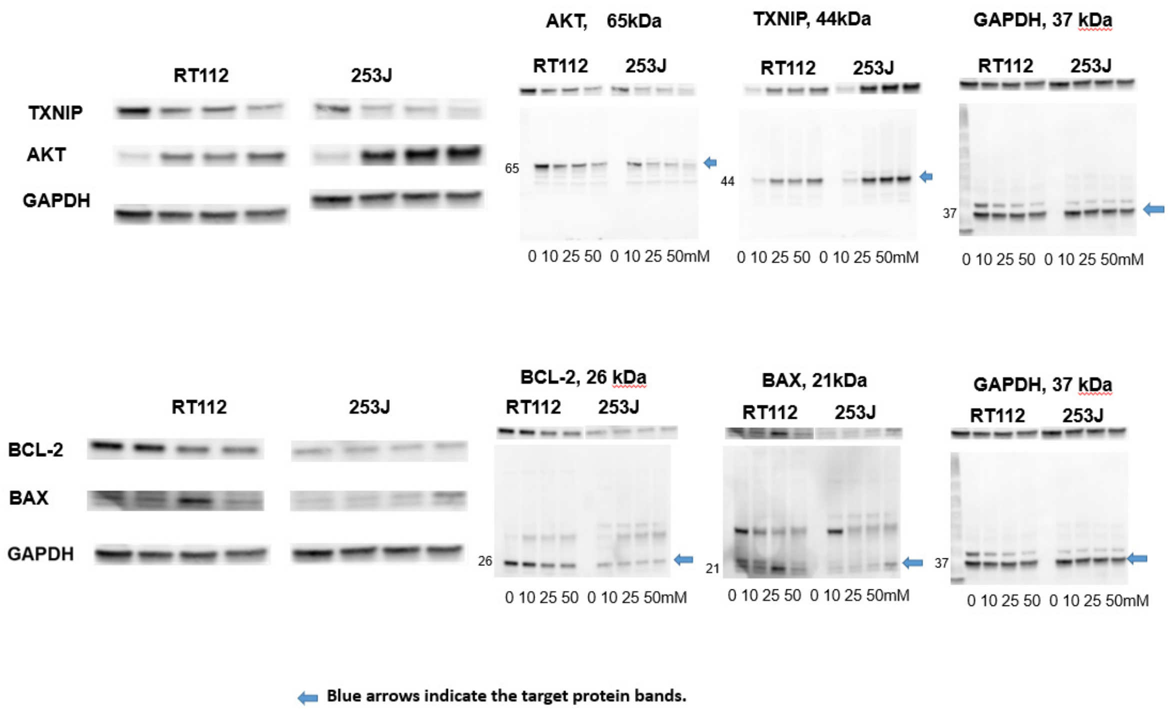

Supplementary Figure S1.Full-length uncropped Western blot images corresponding to Figure 5
